# Childhood Septicemia in Nepal: Documenting the Bacterial Etiology and Its Susceptibility to Antibiotics

**DOI:** 10.1155/2014/452648

**Published:** 2014-12-25

**Authors:** Shamshul Ansari, Hari Prasad Nepal, Rajendra Gautam, Sony Shrestha, Puja Neopane, Brihaspati Rimal, Fuleshwar Mandal, Safiur Rahman Ansari, Moti Lal Chapagain

**Affiliations:** ^1^Department of Microbiology, Chitwan Medical College, Bharatpur, Chitwan, Nepal; ^2^Department of Biochemistry, Chitwan Medical College, Bharatpur, Chitwan, Nepal; ^3^Asian College for Advance Studies, Satdobato, Kathmandu, Nepal

## Abstract

*Introduction*. Children are among the most vulnerable population groups to contract illnesses. The varying microbiological pattern of septicemia warrants the need for an ongoing review of the causative organisms and their antimicrobial susceptibility pattern. Therefore, the objective of this study was to document the bacterial etiology of childhood septicemia and its antibiotic susceptibility profile. *Methods.* Cross-sectional type of study in 1630 suspected patients was conducted at CMCTH from January 2012 to December 2013. Blood samples were collected aseptically for culture. The organisms grown were identified by standard microbiological methods recommended by American Society for Microbiology (ASM) and subjected to antibiotic susceptibility testing by modified Kirby-Bauer disk diffusion method. Methicillin resistance was confirmed using cefoxitin and oxacillin disks methods. *Results.* Septicemia was detected in 172 (10.6%) cases. Among Gram-positive organisms, coagulase negative staphylococci (CoNS) were leading pathogen and *Acinetobacter* spp. were leading pathogen among Gram-negative isolates. Vancomycin, teicoplanin, and clindamycin were the most effective antibiotics against Gram-positive isolates while amikacin was effective against Gram-positive as well as Gram-negative isolates. Methicillin resistance was detected in 44.4% of *Staphylococcus aureus*. *Conclusions*. This study has highlighted the burden of bacterial etiology for septicemia among children in a tertiary care center of central Nepal.

## 1. Introduction

Presence of bacteria within the blood stream, continuous or transient, is known as bacteremia, while the dissemination of bacteria throughout the body with evidence of systemic responses towards microorganism with variable severity is called septicemia [1]. Septicemia is a common cause of pediatric morbidity and mortality [2]. The high mortality rate varies between 30 and 70 percent and depends on several factors including virulence of the pathogen and host factor [3, 4]. Bacteriological culture method to isolate the offending pathogen remains the gold standard method for the diagnosis of bacteremia and septicemia [2, 5]. Organisms isolated from the bloodstream of patients with sepsis vary from area to area [6]. In Nepal, the majority of the bacteremia and septicemia cases are caused by a number of pathogens including coagulase negative staphylococci (CoNS),* Staphylococcus aureus*,* Streptococcus* spp.,* Enterobacter* spp.,* Escherichia coli*,* Klebsiella pneumoniae*,* Salmonella* spp.,* Acinetobacter* spp.,* Citrobacter* spp., and* Pseudomonas* spp. [1, 7, 8].

The rational use of antibiotics for varying microbiological pattern of septicemia in children warrants the need for an ongoing review of the causative organisms and their antimicrobial susceptibility pattern [9, 10]. Uncontrolled use of various potent and broad-spectrum antibiotics has led to emergence of resistant strains which has become a major problem in various intensive care units. Therefore, understanding of common pathogens and their drug sensitivity pattern in a specific region demands the correct use of antibiotics. Due to constantly evolving antimicrobial resistant patterns, there is need for constant antimicrobial sensitivity surveillance. This will help clinicians provide safe and effective empirical therapies, develop rational prescription programs and make policy decisions, and finally assess the effectiveness of all [2].

The results of bacteriological cultures and antibiotic susceptibility tests method are time consuming that can take about 2–4 days, necessitating initial empirical treatment of suspected septicemia. Therefore, knowledge of the epidemiological and antimicrobial susceptibility patterns of common pathogens in a given area helps to inform the choice of antibiotics. In accordance with this, the present study was carried out to document the bacterial etiology of septicemia and its antibiotic sensitivity profile.

## 2. Materials and Methods

A cross-sectional type of study was carried out in bacteriology laboratory of Chitwan Medical College Teaching Hospital (a 600 bed hospital) in Narayani zone of central Nepal from January 2012 to December 2013.

### 2.1. Study Population

A total of 1630 patients during two years of study period between the ages of 1 month and 15 years having clinical features suggestive of septicemia (fever, shortness in breath, weakness, drowsiness, irritability, etc.) were enrolled in this study.

### 2.2. Sample Collection

Two milliliter (mL) of blood samples from early age children and 5 mL of blood samples from late age children were collected aseptically (clearing the skin with 70% alcohol followed by 2% tincture of iodine) by clinicians, trained nurses, or laboratory staff using sterile syringe and needle by venipuncture. Immediately the blood samples were carefully transferred into the blood culture bottle containing 18 mL (if 2 mL blood sample) or 45 mL (if 5 mL blood sample) of Brain Heart Infusion broth to maintain a ratio of 1 : 10 of blood to broth. The blood culture bottles were labeled with the patient's name, age/sex, identification number, date, and time of collection.

### 2.3. Bacteriological Processing

The Brain Heart Infusion broth inoculated with blood was transported to the laboratory and incubated at 37°C in aerobic condition. All the bottles were examined for turbidity, hemolysis, and pellicle formation and subcultures were made on to sheep blood agar, chocolate agar, and MacConkey agar after overnight aerobic incubation. Blood agar and MacConkey agar plates were incubated overnight at 37°C in aerobic atmosphere while chocolate agar plates were incubated overnight at 37°C in 5% carbon dioxide (CO_2_). Culture bottles were observed for turbidity and blind subcultures were performed each day. Final subcultures were done on the 10th day before reporting negative. Growth obtained was examined for colony and Gram-staining characteristics. Conventional biochemical tests were performed and identification of the organism was done by using standard microbiological methods [11]. A purity plate was employed to ensure that the inoculum used for the biochemical tests was pure.

### 2.4. Antibiotic Susceptibility Testing

All the isolates grown were subjected to antibiotic susceptibility testing by modified Kirby-Bauer disk diffusion method in compliance with Clinical and Laboratory Standards Institute (CLSI) guidelines using Mueller-Hinton agar standard media. The inhibition zone standards for antimicrobial susceptibility were considered from tables for interpretative zone diameters of CLSI [12].

Antibiotic disks (HiMedia Laboratories, Pvt. Limited, India) used were as follows: oxacillin (1 *μ*g), cefoxitin (30 *μ*g), vancomycin (30 *μ*g), teicoplanin (30 *μ*g), erythromycin (15 *μ*g), clindamycin (2 *μ*g), penicillin G (10 U), cephalexin (30 *μ*g), cotrimoxazole (25 *μ*g), gentamicin (10 *μ*g), amikacin (30 *μ*g), ofloxacin (5 *μ*g), cefixime (5 *μ*g), cefotaxime (30 *μ*g), ceftazidime (30 *μ*g), piperacillin (100 *μ*g), piperacillin-tazobactam (100/10 *μ*g), carbenicillin (100 *μ*g), amoxycillin (10 *μ*g), and nalidixic acid (30 *μ*g).

*Staphylococcus aureus* ATCC 25923 and* Escherichia coli* 25922 were used as control organisms for antibiotic sensitivity testing.

### 2.5. Identification of Methicillin Resistance in* Staphylococcus aureus*

Identification of methicillin resistant* Staphylococcus aureus* (MRSA) strains was carried out by using oxacillin (1 *μ*g) and cefoxitin (30 *μ*g) disks. Plates were incubated at 35°C. Plates containing oxacillin disk were read following a 24-hour incubation period. The diameter of the zone of inhibition (ZOI) of growth was recorded and interpreted as susceptible or resistant according to the criteria of CLSI.* Staphylococcus aureus* isolates were deemed methicillin resistant when the ZOI was ≤10 mm with the oxacillin disk or ≤21 mm with the cefoxitin disk [13].

### 2.6. Ethical Aspects

Verbal consent in local language for this study was taken from the guardians of participating children. This study was approved by the Institutional Review Committee of Chitwan Medical College, Bharatpur, Nepal.

### 2.7. Data Analysis

Statistical analysis was performed using SPSS-11.5 version. Association of septicemia with gender and type of causative agents were assessed by using the chi-square test and others. *P* values < 0.05 were considered statistically significant.

## 3. Results

### 3.1. Age and Sex-Wise Distribution of Cases

During two years of study period, 1630 children aged between 1 month and 15 years were enrolled, from whom positive growth of bacteria was obtained in 172 cases (10.6%). Of total enrolled cases, 1016 (62.3%) were males and 614 (37.7%) were females, whereas, out of total positive cases, 94 (54.6%) were males and 78 (45.4%) were females which was found to be statistically significant (*P* = 0.028). Suspected cases and positive cases were more or less equal in each age group (Table 1).

### 3.2. Distribution of Bacterial Isolates

Gram-positive as well as Gram-negative organisms were isolated from the blood samples. Gram-positive organisms constituted 47.7% while Gram-negative organisms constituted 52.3% but the difference was not found to be statistically significant (*P* = 0.388). Of total positive cases, CoNS were the most common isolates (39.5%) followed by* Acinetobacter* spp. (17.5%). 44.1% (30/68) of the CoNS positive cases belonged to the age group of 1–11 months (Table 2).

### 3.3. Antibiotic Resistance Pattern

Vancomycin and teicoplanin were found to be hundred percent effective against Gram-positive isolates. Amikacin was found to be very good alternative antibiotic for Gram-positive as well as Gram-negative isolates with very minor resistance to it. Most of the Gram-positive isolates were resistant to erythromycin, cotrimoxazole, and *β*-lactam antibiotics. Most of the tested antibiotics were not effective against Gram-negative isolates and nearly all isolates were resistant to ampicillin. Methicillin resistance was observed in 35.5% of CoNS and 44.4% of* Staphylococcus aureus* isolates whereas nalidixic acid resistance (NARS) was observed in 33.3% of* Salmonella* spp. (Table 3).

## 4. Discussion

Bacterial infections are major causes of morbidity and mortality in children. The detection, identification, and susceptibility testing of a causative species of bacteremia are essential for the proper management of the patient. In this study, among 1630 children aged between 1 month and 15 years enrolled, positive growth of bacteria was found in 172 cases (10.6%). High culture positivity rates have been reported by investigators from other countries, 44.9% from Nigeria [2], 44.8% from Jordan [14], and 22.9% from India [5]. Various factors affect the rate of isolation of organisms from the blood. These include the degree of bacteremia, prior antibiotic therapy, presence of fastidious organisms, the collection time and process, the ratio of amount of blood collected to volume of liquid broth, and the prolonged storage or delay prior to plating on solid media [15]. However, our isolation rate is higher than the rate of bacterial growth (4.2%) reported from Kathmandu, Nepal [7], where most of the cases were referred from other centers and probably had received antibiotics prior to blood collection in the study center.

In current study, the bacterial sepsis was suspected more in male children (62.3%) than in female children (37.7%) and the higher rate of bacterial isolation in males (54.6%) compared to females (45.4%) seen in this study was found to be statistically significant (*P* < 0.05). Our result is in accordance with Karki et al. from Nepal [7] and Nimri et al. from Jordan [14], who have also reported higher bacterial growth in male patients than female patients. The higher positivity rate of septicemia in male children may be because of several factors. Firstly, male children are affected more often and more severely by infectious diseases and their immune systems respond less effectively to vaccine. Secondly, the testosterone can also suppress the immunity in male children [16]. Thirdly, females have more powerful immune system than males. The production of estrogen by females can have a beneficial effect on the innate inflammatory response against bacterial pathogens [17].

The causative organisms vary from place to place. In this study, Gram-positive as well as Gram-negative organism was isolated from the blood samples. Gram-positive organisms constituted 47.7% while Gram-negative organisms constituted 52.3% but the difference was not found to be statistically significant (*P* > 0.05). The various causative agents of childhood septicemia in this study were CoNS (39.5%) followed by* Acinetobacter* spp. (17.5%),* Pseudomonas aeruginosa* (9.9%),* Enterobacter* spp. (7.6%),* Staphylococcus aureus* (5.2%),* Escherichia coli* (5.2%),* Salmonella* spp. (5.2%),* Klebsiella pneumoniae* (4.6%),* Enterococcus* spp. (3.0%), and* Citrobacter* spp. (2.3%). CoNS as the leading cause of childhood septicemia followed by* Enterococcus* spp.,* Escherichia coli*,* Klebsiella pneumoniae*, and* Pseudomonas aeruginosa *were also reported by Nimri et al. from Jordan [14].

Coagulase negative staphylococci (CoNS) were found to be the most common etiology of septicemia in the present study. Our result is in agreement with other studies that reported CoNS as the most common bacteria isolated from children with sepsis [3]. Coagulase negative staphylococci were reported to have emerged as a major cause of nosocomial infections [18]. They are part of normal flora and their presence in blood cultures might indicate catheter and medical device-related sepsis or a contaminant of blood cultures [19]. The interpretation of their presence is a major concern for clinicians and clinical microbiology laboratories. The decision for therapy relies mostly on the observation of sepsis symptoms and the number of positive blood cultures. However, the criteria of multiple blood cultures could not be applied in this study in early age pediatric patients who could not undergo multiple venipuncture.* Acinetobacter* spp. was isolated from 17.5% of cases and was the second most common isolated organism.

Though penicillin is a primary drug against Gram-positive organisms, such isolates in our study exhibited 80% resistance to it. This result concurs with the report of Karki et al. from Nepal, who also noticed that* Staphylococcus *spp., the important Gram-positive organisms, were the least susceptible to penicillin [7]. All of the Gram-positive isolates were susceptible to vancomycin and teicoplanin whereas clindamycin was effective against more than 70% isolates.

Despite widespread use of aminoglycosides (gentamicin and amikacin), development of resistance to this class of antibiotics remained low compared to other antimicrobial agents. Amikacin was found to be a very good alternative for Gram-positive as well as Gram-negative isolates with very minor isolates being resistant to it while nearly half of the isolates were resistant to gentamicin. This observation was confirmed by the reports that the majority of Gram-positive as well as Gram-negative blood stream isolates were susceptible to amikacin [20–22]. Resistance to gentamicin has recently increased and it may be due to overuse or misuse of this antibiotic in patients [23, 24].

Resistance to macrolide (erythromycin) is also increasing; more than 50% of isolates were resistant to erythromycin and most of the Gram-positive isolates were resistant to cotrimoxazole in this study. Similar result indicating high resistance to erythromycin and cotrimoxazole was also documented by Rathod et al. from India [25]. While fluoroquinolone is predominantly a Gram-negative drug, it does have activity against Gram-positive organisms also. As a consequence of low cost and easy availability, there has been indiscriminate use of ciprofloxacin in Nepal. We identified resistance rate ranging from 20% in* Enterococcus* spp. to 61.5% in* Enterobacter* spp.

Third generation cephalosporins were also less effective against Gram-negative isolates in this study. The resistance of 15.4% was exhibited by* Enterobacter* spp. and 77.3% by* Acinetobacter* spp. In a similar study conducted by Omoregie et al. in Nigeria, 100% of Gram-negative isolates were resistant to third generation cephalosporins [13]. Ceftriaxone and ceftazidime are being used without laboratory guidance, especially as coverage antibiotic during surgery and as blind antibiotic in emergencies, which perhaps has resulted in bacterial resistance to this drug. Amoxycillin was also not effective against Gram-negative isolates, 77% to 100% of Gram-negative isolates were resistant to amoxycillin. Omoregie et al. from Nigeria have also observed that nearly all Gram-negative isolates were found to be resistant to amoxicillin [13].

Nowadays, the methicillin resistance in CoNS and* Staphylococcus aureus* (MRSA) is posing a great challenge to the treatment narrowing the regimen options for these resistant bugs. Prior antibiotic use is the most common risk factor for colonization and infection with MRSA. In this study, 35.5% of CoNS and 44.4% of* Staphylococcus aureus* isolates were found to be resistant to methicillin. This result corroborates with 30.7% MRSA observed in children suffering from bacteremia by Saravanan et al. in India [26]. Two different methods were employed for the detection of MRSA. The cefoxitin disk method detected 44.4% of MRSA cases while the oxacillin disk method detected 33.3% of MRSA. According to CLSI the cefoxitin disk test is comparable to the oxacillin disk test for the prediction of mecA-mediated resistance to oxacillin [27]. The cefoxitin disk test is easier to read and thus is the preferred method. Besides, cefoxitin is an inducer of the mecA gene.

The emergence of quinolone resistance in the most common* Salmonella* serotype worldwide is a serious public health concern. Resistance to nalidixic acid has been associated with reduced efficacy of fluoroquinolones such as ciprofloxacin and ofloxacin [28, 29]. In current study, 33.3% of* Salmonella* spp. were nalidixic acid resistant (NARS).

There was no attempt to isolate anaerobic bacteria in this study although they might have been the cause of bacteremia and septicemia in some of the cases where no aerobic bacteria were detected in blood cultures. Anaerobic bacteria were reported to constitute 18% of the total number of isolates from blood [30].

## 5. Conclusions

This study has highlighted the burden of bacterial etiology for septicemia among children in a tertiary care center of central Nepal. However, since the spectrum of pathogens, incidence of diseases, and antimicrobial susceptibility change over time and places, the data should be monitored continuously to allow an appropriate clinical response and healthcare planning. This study highlights the variable nature of antibiotic susceptibility patterns. Therefore, it is advisable to continuously evaluate the resistance pattern of isolates so as to make a rational use of antibiotics.

The present study identified the burden of MRSA infection among septicemic children. Regular monitoring of antibiotic susceptibility pattern of MRSA and selection of a definite antimicrobial agent may be helpful for reducing the incidence of MRSA infections in septicemia in children. It is also important for clinicians to be aware of the existence of the bacterial strains showing decreased fluoroquinolone susceptibility.

## Figures and Tables

**Table 1:** Distribution of total and positive cases.

Age	Total cases (*n* = 1630)		Positive cases (*n* = 172)		
	Male	Female	Male	Female	*P* value
1–11 months	210	136	28	29	0.028
1–5 years	295	187	26	22	
6–10 years	269	144	18	15	
11–15 years	242	147	22	12	

Total	1016 (62.3%)	614 (37.7%)	94 (54.6%)	78 (45.4%)	

**Table 2:** Distribution of bacterial isolates.

Isolates	1–11 months	1–5 years	6–10 years	11–15 years	Total (%)	*P* value
Gram-positive	**34**	**19**	**17**	**12**	**82 (47.7)**	**0.388**
Coagulase negative staphylococcus (CoNS)	30	16	14	8	68 (39.5)	
*Staphylococcus aureus *	3	2	1	3	9 (5.2)	
*Enterococcus *spp.	1	1	2	1	5 (3.0)	
Gram-negative	**23**	**29**	**16**	**22**	**90 (52.3)**	
*Acinetobacter *spp.	8	8	6	8	30 (17.5)	
*Citrobacter *spp.	0	3	0	1	4 (2.3)	
*Enterobacter *spp.	2	8	1	2	13 (7.6)	
*Pseudomonas aeruginosa *	8	4	3	2	17 (9.9)	
*Klebsiella pneumoniae *	1	2	3	2	8 (4.6)	
*Escherichia coli *	3	2	2	2	9 (5.2)	
*Salmonella *spp.	1	2	1	5	9 (5.2)	

Total (%)	57 (33.1)	48 (27.9)	33 (19.2)	34 (19.8)	172 (100)	

**Table 3:** Antibiotic resistance profile of isolates.

Isolated organisms	Rate of resistance to different antibiotics tested																			
	OX	FOX	VAN	TEI	E	CD	P	CFX	COT	G	AK	OF	CFM	CTX	CAZ	PI	PIT	CAR	AMX	NA
Coagulase negative* Staphylococcus *spp.	29.4	35.3	0	0	51.5	26.5	88.2	80.9	67.6	55.9	2.9	32.4	X	X	X	X	X	X	X	X
*Staphylococcus aureus *	33.3	44.4	0	0	55.6	22.2	88.9	77.8	44.4	44.4	0	22.2	X	X	X	X	X	X	X	X
*Enterococcus *spp.	0	X	0	0	60.0	0	100	X	X	60.0	20.0	20.0	X	X	X	X	X	X	X	X
*Acinetobacter *spp.	X	X	X	X	X	X	X	X	56.7	26.7	10.3	20.0	76.7	66.7	77.3	60.0	43.3	X	83.3	X
*Pseudomonas aeruginosa *	X	X	X	X	X	X	X	X	X	52.9	11.7	35.3	58.8	70.6	58.8	76.5	52.9	41.2	100	X
*Citrobacter *spp.	X	X	X	X	X	X	X	X	50.0	75.0	0	25.0	0	50.0	50.0	50.0	25.0	X	100	X
*Enterobacter *spp.	X	X	X	X	X	X	X	X	69.2	61.5	23.0	61.5	69.2	15.4	23.0	61.5	38.5	X	77.0	X
*Klebsiella pneumoniae *	X	X	X	X	X	X	X	X	62.5	50.0	0	37.5	50.0	37.5	37.5	62.5	37.5	X	100	X
*Escherichia coli *	X	X	X	X	X	X	X	X	77.8	66.7	11.1	33.3	22.2	55.5	66.7	77.8	55.5	X	77.8	X
*Salmonella *spp.	X	X	X	X	X	X	X	X	44.4	22.2	0	33.3	33.3	55.5	44.4	55.5	22.2	X	88.9	33.3

Note: OX: oxacillin, FOX: cefoxitin, VAN: vancomycin, TEI: teicoplanin, E: erythromycin, CD: clindamycin, P: penicillin G, CFX: cephalexin, COT: cotrimoxazole, G: gentamicin, AK: amikacin, OF: ofloxacin, CFM: cefixime, CTX: cefotaxime, CAZ: ceftazidime, PI: piperacillin, PIT: piperacillin-tazobactam, CAR: carbenicillin, AMX: amoxycillin, NA: nalidixic acid, X: not tested.
